# Can flow proneness be protective against mental and cardiovascular health problems? A genetically informed prospective cohort study

**DOI:** 10.1038/s41398-024-02855-6

**Published:** 2024-03-13

**Authors:** Emma Gaston, Fredrik Ullén, Laura W. Wesseldijk, Miriam A. Mosing

**Affiliations:** 1 Melbourne School of Psychological Sciences, Faculty of Medicine, Dentistry, and Health Sciences, University of Melbourne, Melbourne, Australia; 2 Department of Cognitive Neuropsychology, Max Planck Institute for Empirical Aesthetics, Frankfurt, Germany; 3 Department of Neuroscience, Karolinska Institutet, Stockholm, Sweden; 4 Department of Psychiatry, Amsterdam UMC, University of Amsterdam, Amsterdam, Netherlands; 5 Department of Medical Epidemiology and Biostatistics, Karolinska Institutet, Stockholm, Sweden

**Keywords:** Depression, Human behaviour, Diagnostic markers, Bipolar disorder, Schizophrenia

## Abstract

Flow is a phenomenon where one experiences optimal challenge, marked by an intense, effortless, and rewarding concentration on a task. Past research shows that flow proneness is associated with good mental and cardiovascular health. However, this research has been primarily cross-sectional, based on self-report data, and has not controlled for potential confounding effects of neuroticism. In a large, longitudinal twin sample (*N* = 9361), we used nationwide patient registry data to test whether flow proneness predicted registry-based diagnoses of depression, anxiety, schizophrenia, bipolar disorder, stress-related disorders, or cardiovascular diseases. We used survival analyses taking time to diagnosis into account to test if (a) there is a relationship between flow proneness and health diagnoses over time, (b) neuroticism confounds this relationship, and (c) the relationship remains present within discordant monozygotic twin pairs (*N* = 952), thereby controlling for genetic and shared environmental confounding. Individuals with higher flow proneness had a decreased risk of receiving diagnoses for depression (16%; CI [14%, 18%]), anxiety (16%; CI [13%, 18%]), schizophrenia (15%; CI [4%, 25%]), bipolar (12%; CI [6%, 18%]), stress-related (9%; CI [9%, 12%]), and cardiovascular disorders (4%; CI [1%, 8%]). When controlling for neuroticism, higher flow proneness still decreased the risk of depression (6%; CI [3%, 9%]) and anxiety diagnoses (5%; CI [1%, 8%]). Monozygotic twins who experienced more flow than their co-twin had a lower risk for depression (16%; CI [5%, 26%]) and anxiety (13%; CI [1%, 24%]), though only the association with depression remained significant when also controlling for neuroticism (13%; CI [1%, 24%]). Findings are in line with a causal protective role of flow experiences on depression and potentially anxiety and highlight that neuroticism and familial factors are notable confounding factors in observed associations between flow proneness and health outcomes.

## Introduction

Flow is the psychological state of optimal challenge, marked by an intense and focused concentration on the present task, reduced self-consciousness, a sense of control, a merging of action and awareness, altered time perception, and experiencing a task as intrinsically rewarding [1]. The phenomenon was first described by Csikszentmihalyi in the 70 s [2]. Jackson and colleagues determined and first measured some components of flow state [3–5], with flow conceptualized as a state incorporating nine dimensions (including having a sense of control and unambiguous feedback, among others) [6].

Since then, research has shown there are large individual differences in proneness to experience flow (flow proneness; FP) [7, 8]. From state flow characteristics emerged the concept of the autotelic personality; that is, someone with a disposition that actively seeks and experiences flow more often than others [9]. The autotelic individual is prone to flow, and in this way, FP can be both a state (related to circumstance) [2, 10] and a stable trait factor [11], possessed by everyone on a continuum [7].

FP has received much publicity in recent years, partly due to the notion that it may be beneficial for mental and somatic health. For example, FP is positively associated with higher self-esteem, self-concept, and self-efficacy [3, 5, 12]; greater life satisfaction [12, 13]; active coping strategies [12]; intrinsic motivation [3, 8]; and psychological well-being [12]. Some studies report a negative association between FP and anxious symptomatology (e.g., [3, 12, 14–17]). Furthermore, research has shown that flow experiences can ameliorate anxious and sad moods (e.g., [12, 18]) and may prevent rumination or future worrying through merging action and awareness [19, 20], indicating that FP may be preventative of depression, anxiety, and stress disorders. It has been suggested that flow states may be accompanied by a co-activation of parasympathetic and sympathetic branches of the autonomous nervous system [21, 22], which may reduce stress [23] and anxiety as posited by flow theory [2].

There may also be an association between FP and decreased risk of physical ill-health [24, 25]. Though this is not an entirely consistent picture, research supports that parasympathetic co-activation would be better for long-term cardiovascular health than consistently stressful states with parasympathetic deactivation [26]. If flow’s properties do really reduce stress, it may over time also be related to cardiovascular disease, given the disease is closely related to stress [27].

Above associations are commonly interpreted as evidence for a protective causal effect of FP upon mental and somatic health and although little is understood about the amenability of flow experiences, the first training programs have been introduced claiming to enhance flow (e.g., www.flowresearchcollective.com/training). However, apart from largely being cross-sectional, earlier studies do not account for reverse causality (i.e., mental health problems resulting in lower flow proneness) and confounding factors that underlie both health and FP, such as shared genetic influences, environmental factors, and personality and therefore do not allow for a causal interpretation. This highlights the importance of rigorous research on the nature of such associations.

Mosing et al. [28] found that individual variation in FP is moderately heritable, with estimates between 29–35%. Even though this is somewhat lower than most personality characteristics, which are ~40% heritable [29], this shows that genetic factors play a role in individual differences in FP. Similarly, it is well established that genetic factors influence individual differences in somatic and mental health [28]. Therefore, it is likely that shared genetic influences may at least partly explain observed associations between FP and health.

To our knowledge, only one study has used a genetically informative design to adjust for shared familial factors when exploring FP and health [30, 31]. Mosing and colleagues found the relationship between FP and burnout and depressive symptoms to be partially confounded by familial factors (genetic and rearing environment). Nonetheless, after taking familial factors into account, the relationship between FP and burnout and depressive symptoms remained, strengthening the case for a causal relation. This study, however, used a self-reported measure of mental health and cross-sectional data rather than registry-based health diagnoses including a date, which allows for analyzing the time to diagnoses (i.e., longitudinal time-to-event analyses).

Importantly, FP is also linked to personality [32, 33], and has shown to be positively associated with conscientiousness, and negatively associated with neuroticism [7], which is a trait that encompasses irritability, anger, depression, anxiety, hostility, sensitivity, and worry [34]. Those high in neuroticism are more susceptible to stress and mental health problems [35–37] as well as cardiovascular diseases and other somatic conditions [38, 39]. Neuroticism may therefore confound observed relationships between FP and health, but no studies to date have explored the associations between FP and health whilst also considering neuroticism.

In the present study, we investigate the relationship between FP and lifetime mental and somatic health diagnoses extracted from nationwide patient registries utilizing longitudinal and genetically informative data from ~9500 Swedish twins. In addition, we examine whether any observed associations remain after controlling for neuroticism. Finally, making use of discordant twin data, we explore whether observed associations between FP and mental and somatic health remain when adjusting for familial confounding factors [40], strengthening causal inferences.

## Methods and materials

### Participants

In 2012-2013, the STAGE cohort of the Swedish Twin Registry [41–44] was invited to complete a web survey containing, among other questions, items measuring personality traits, mental health problems and FP. In total, 11,543 twin individuals participated and data on FP were available for 9,366 individuals. Further details regarding the survey can be found in earlier studies [30, 45–47].

We linked data on date and type of health diagnoses from the Swedish National Patient Registries (NPR) with the twin data from the STAGE cohort. The NPR includes an inpatient registry (with hospitalizations from 1964 and sufficient coverage since 1977) and an out-patient registry (with full coverage since 2001). We excluded five participants as their data could not be linked with health records from the NPR. The final sample included 9361 twin individuals (58.9% females) including 2058 complete twin pairs: 1010 dizygotic and 984 monozygotic twin pairs (and 64 complete twin pairs with an unknown zygosity), see Table 1.

**Table 1:** Descriptive Characteristics of Participants.

Characteristic	*N* individuals (%)	*M (SD)*
Monozygotic	3577 (38.2)	
Dizygotic	5784 (61.2)	
Age^a^		40.12 (7.74)
Sex (Female)	5511 (58.9)	
Flow Proneness Score		26.04 (3.2)
Education Level Available	7550 (80.7%)	6.42 (2.02, range 1-10)
BFI-44 Score Available	8986 (95.99)	2.41 (0.67)

Diagnoses	*N* (%)	*M (SD)* age of first diagnosis
Depression	498 (5.3)	35.02 (9.04)
Anxiety	432 (4.6)	34.53 (9.55)
Schizophrenia	27 (0.3)	31.78 (9.60)
Bipolar disorder	91 (1.0)	34.00 (9.42)
Cardiovascular diseases	445 (4.8)	42.04 (10.43)
Stress disorders	302 (3.2)	36.51 (9.60)

Note. *N* total number of cases, *M* grand mean*, SD* standard deviation, *BFI-44* Big Five Inventory.

^a^Age at the time of data collection (2012).

This study received approval from the Regional Ethics Review Board in Stockholm (Dnr 2011/570-31/5, 2012/1107-32, 2018/866-32). Analyses were preregistered at *As Predicted* (#65610); https://aspredicted.org/blind.php?x=ev7qa5.

### Measures

#### Flow proneness

The Swedish Flow Proneness Questionnaire (SFPQ) measures individuals’ flow during work, maintenance, and leisure, with higher scores indicating a higher frequency of flow experiences [7]. See Appendix A for all items of the SFPQ. Global FP was calculated as the mean score of the three subscales (i.e., work, maintenance, and leisure), or the mean score of two subscales in case a score on one of the scales was missing. The Cronbach alpha reliability was 0.82 for the work, maintenance, and leisure scale in this sample. For more psychometric data see [7].

#### Somatic and mental health

Registry-based diagnoses (i.e., diagnosis type and date) for depression, anxiety, schizophrenia, bipolar disorder, stress-related disorders, and cardiovascular diseases were obtained from NPR mental and physical diagnoses. Appendix B indicates the ICD codes for the specific diagnoses included in the analysis from 1977 until present.

#### Neuroticism

Neuroticism was measured with the Swedish translation of the Big Five Inventory (BFI; [48]). The full scale includes 44 items, of which 8 are used to calculate neuroticism scores. See Appendix C for the items of this subscale. Items were answered in Likert fashion, from 1 – “strongly disagree” to 5 – “strongly agree”. The Cronbach alpha reliability for the scale was 0.83 in this sample.

### Analyses

#### The association between flow proneness and the risk of health diagnoses

Survival analyses (in this case Cox proportional hazard regressions) were conducted to explore the effect of FP on mental and physical health diagnoses. Survival analyses model the outcome variable as the time until the occurrence of an event. In this case, the number of months of survival (age) from either the age of twelve (to exclude children) or age in 1977 until date of diagnosis or date of censoring (death or end of follow-up in January 2017) was used as the time scale. Thus, age was accounted for in every survival model. All analyses were conducted in Stata (version 16.1; [49]), and sex was included as a covariate in every model. As only one hypothesis (a significant relationship between FP and health diagnoses) was tested, a conventional *p*-value cut-off of alpha = 0.05 was used as indicator of significance, with actual *p*-values (rather than significance levels) reported.

Hazard Ratios (HRs) representing the effect of a one-unit increase in FP on the probability for receiving a diagnosis were obtained from the Cox proportional hazard regressions. Separate survival models were fit for each diagnosis. Given the assumption of independent data points in regression analyses, the robust standard error estimator was used to correct for relatedness in the twin sample [50]. The proportional hazards assumption was tested with Schoenfeld residuals for each survival model [51].

Although flow proneness is a relatively stable trait [7, 13, 26], to exclude reverse causality, sensitivity analyses were run in a sub-sample where those who received the respective diagnoses before 2012 were removed to ensure that FP was measured before a diagnosis was obtained. Sub-samples for each analysis ranged between *N* = 8987–9161.

#### The confounding effect of neuroticism in the flow proneness and health relationship

A linear regression model was fit to estimate the association between phenotypic neuroticism and FP. Sex and age were included as covariates under the assumption that these variables may impact neuroticism levels [52] and FP [8, 28]. Then, survival analyses as described above were repeated with phenotypic neuroticism as a covariate to test whether neuroticism levels (partially) explained the association between FP and the risk of diagnoses. Again, the robust standard error estimator for clustered observations was used to correct for the absence of independence in data points.

#### Possible causal effects of flow proneness on the risk of health diagnoses

To test whether the associations between FP and mental and somatic health outcomes are in line with a causal hypothesis we conducted co-twin control analyses [40]. Because monozygotic twins are genetically identical and share their rearing environment, studying differences in health outcomes in pairs that are discordant for FP allows us to test whether FP still influences health outcomes when genetics and familial environment are kept constant. If FP truly lowers the risk of disease, we expect twins with higher FP to have a lower risk for health problems than their co-twin with lower FP. We fit within-pair conditional Cox regression models separately for each health diagnoses to estimate within-pair HRs. These were stratified by pair identifier to compare twins within pairs against each other. However, only twins discordant for exposure (i.e., different FP scores) and outcome (i.e., one or both receive a diagnoses at different points in time) contribute to these co-twin control analyses (see N for each analyses in the Tables). In additional analyses, we added neuroticism as a covariate into the co-twin control analyses to test for whether differences in neuroticism within a twin pair can account for the effect of FP on health diagnoses.

## Results

Table 1 presents the sample characteristics of participants with linked registry data. Depression and anxiety were the most common conditions, while bipolar disorder and schizophrenia were diagnosed at the lowest rates. A linear regression of FP on age and sex showed that FP differed significantly by age, *b* = 0.04, *t*(7303) = 9.52, *p* < 0.001, 95% CI [0.03, 0.05], and sex, *b* = 0.46, *t*(7303) = 6.60, *p* < 0.001, 95% CI [0.32, 0.59], with females and older individuals experiencing more flow.

### The association between flow proneness and the risk of health diagnoses

**Fig. 1:**
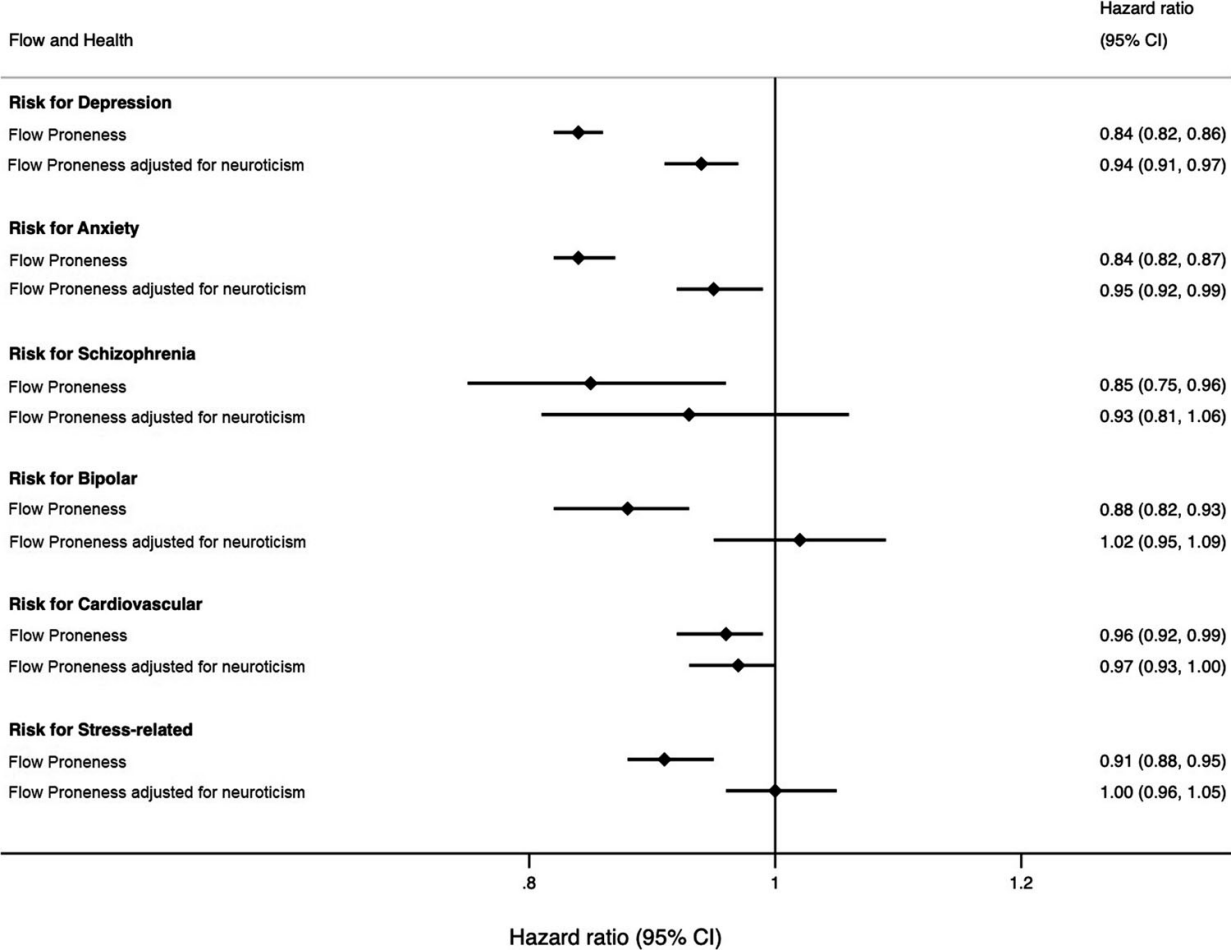
Forest plot with hazard ratios demonstrating the associations between flow proneness and risk of diagnoses unadjusted and adjusted for neuroticism.

HRs from survival models investigating the longitudinal relationship between FP and diagnoses (Fig. 1, *unadjusted*) indicated that each unit increase in flow resulted in a 16% lower risk for receiving a diagnosis of depression (CI [14%, 18%]) and anxiety (CI [13%, 18%]), a 15% lower risk of schizophrenia (CI [4%, 25%]), a 13% lower risk of bipolar disorder (CI [7%, 18%]), a 10% lower risk of stress disorders (CI [5%, 12%]) and a 5% lower risk of cardiovascular diseases (CI [1%, 8%]). See Appendix D (Table 1), for tables of all HRs obtained from the models.

### The confounding effect of neuroticism in the flow proneness and health relationship

Neuroticism was significantly associated with FP, *b* = −2.05, *t*(7047) = −41.05, *p* < 0.001, 95% CI [−2.15, −1.95], accounting for 17.56% of the variance in FP. With a correlation below 0.8, (*r* = −0.42, *p* < 0.001; [53]) and a mean variance inflation factor of 1.23, we assume non-collinearity was not violated [54]. Note that correlations between FP and neuroticism on the item level ranged between −0.20 (BFI item 29) and −0.38 (BFI item 4).

When adding phenotypic neuroticism to the survival models, neuroticism was associated with the risk of obtaining all diagnoses (see Appendix D, Table 1). Further, the effect size of the HRs for FP significantly decreased for depression, anxiety, bipolar and stress-related disorders (HRs were closer to one and non-overlapping confidence intervals), indicating confounding by neuroticism (Fig. 1, *adjusted for neuroticism*). When adjusting for neuroticism, depression and anxiety remained significantly negatively associated with FP. For every additional point on the FP scale, the risk of depression diagnosis decreased 6% (CI [3%, 9%]) and anxiety 5% (CI [1%, 8%]). However, associations between FP and schizophrenia, bipolar disease, cardiovascular disease, and stress-related disorders, respectively, became non-significant when adjusting for neuroticism. Schoenfeld residuals indicated that there was no deviation from the proportional hazards assumption for any of the survival models; all *p*-values > 0.01 [51].

### Testing for reverse causality in the flow proneness and health relationship

**Fig. 2:**
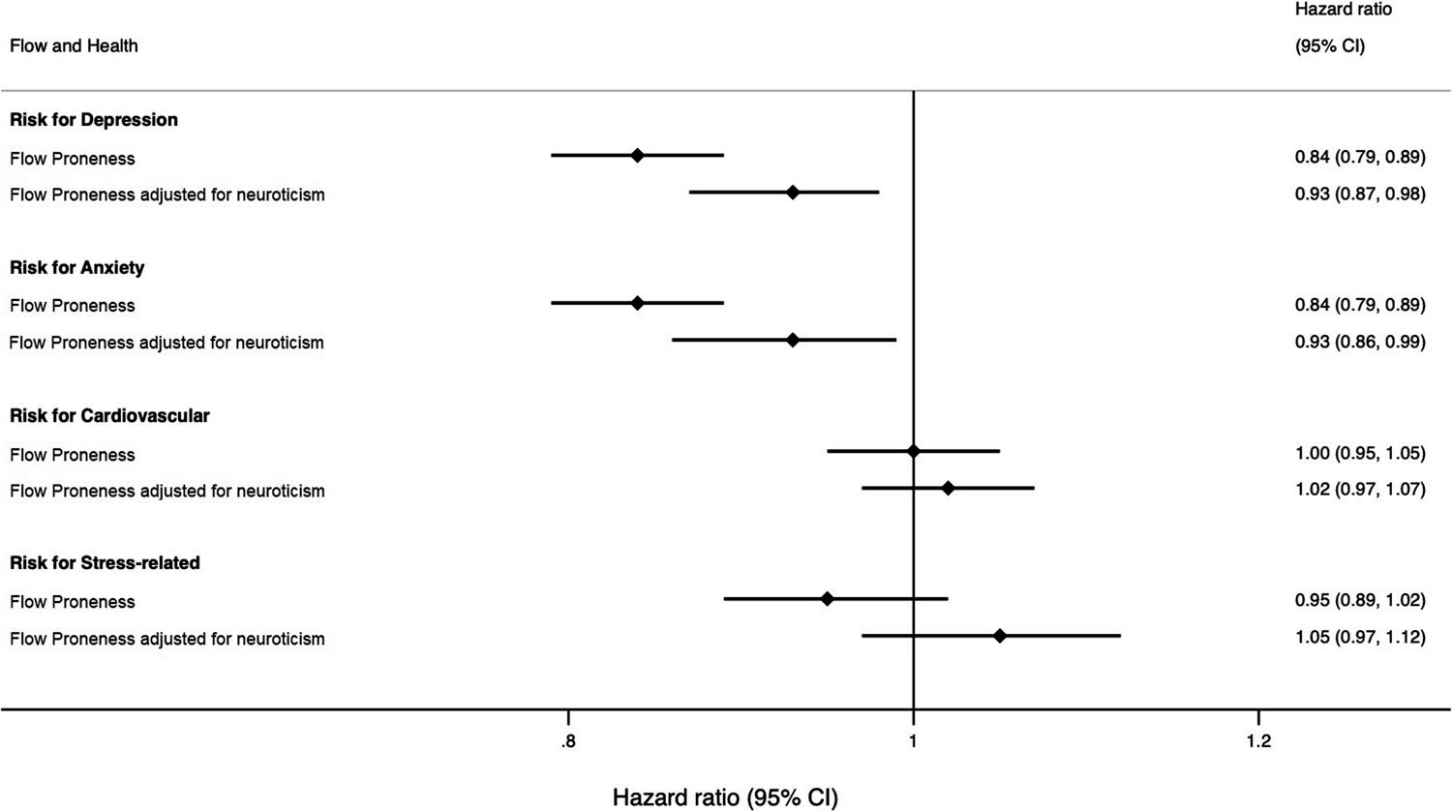
Forest plot with hazard ratios demonstrating the association between higher flow proneness and risk of diagnosis post-2012. Numbers of those diagnosed with respective conditions in these analyses are as follows: *N*_*depression*_ = 126; *N*_*anxiety*_ = 124; *N*_*cardiovascular*_ = 185; *N*_*stress*_ = 103. After adjusting for neuroticism: *N*_*depression*_ = 121; *N*_*anxiety*_ = 115; *N*_*cardiovascular*_ = 177; *N*_*stress*_ = 99. There were too few cases post 2012 to perform sensitivity analyses for schizophrenia and bipolar diagnoses.

Results remained similar in sensitivity analyses only including diagnoses first obtained after 2012 (Fig. 2), with higher FP being associated with a reduced risk for depression (16%, CI [11%, 21%]) and anxiety (16%, CI [11%, 21%]). These associations remained significant, even after adjusting for neuroticism, with higher FP associated with a 7% reduced risk of depression (CI [2%, 13%]), and 7% reduced risk of anxiety (CI [1%, 14%]). This suggests that reverse causation is an unlikely explanation for the observed associations. Associations disappeared for cardiovascular and stress disorders, suggesting reverse causality may have an impact here. Due to too few individuals diagnosed with schizophrenia (*N* = 2) and bipolar disorder *(N* = 7) after 2012, we were not able to conduct those sensitivity analyses for these two disorders. See Appendix D (Table 2), for tables of all HRs obtained from the models.

### Possible causal effects of flow proneness on the risk of health diagnoses

**Fig. 3:**
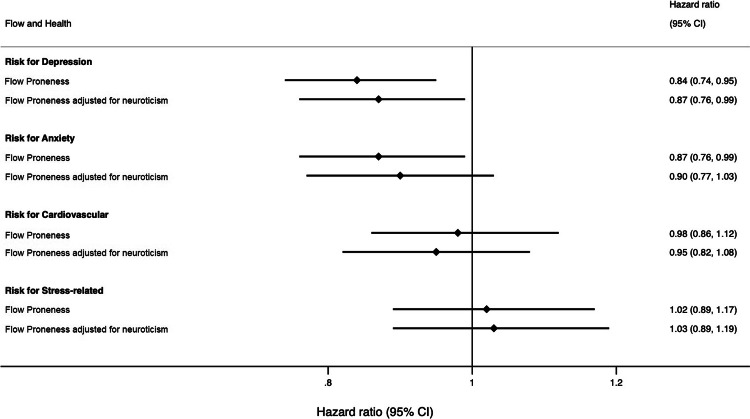
Co-twin control analyses forest plot with hazard ratios demonstrating the associations between higher flow proneness and risk of diagnosis. Numbers of discordant twin pairs on being diagnosed in these analyses are as follows: *N*_*depression*_ = 97; *N*_*anxiety*_ = 79; *N*_*cardiovascular*_ = 68; *N*_*stress*_ = 57. After adjusting for neuroticism: *N*_*depression*_ = 96; *N*_*anxiety*_ = 76; *N*_*cardiovascular*_ = 63; *N*_*stress*_ = 55.

The co-twin control analyses revealed that higher FP was associated with a decreased risk of depression after accounting for common genetics (including age and sex) and rearing environment. A 16% lower risk of depression (CI [5%, 26%]) for each additional unit in FP score was found within pairs (Fig. 3) – mirroring the association observed in the full sample. After accounting for neuroticism, every point higher in FP was significantly associated with a 13% lower risk of depression (CI [1%, 24%]), suggesting that systematic differences in neuroticism cannot explain this association.

FP was no longer significantly associated with any of the other diagnoses, although the effect size for anxiety (13% reduced risk for every point higher in FP (CI [1%, 24%])); 10% after adjusting for neuroticism (CI [-3%, 23%]) remained similar to that found in the full sample, suggesting that there may be a true effect that we did not have the power to detect. Schizophrenia and bipolar disorder analyses could not be run due to too few cases.

See Appendix D (Table 3), for tables of all HRs obtained from the models.

## Discussion

We investigated the relationship between FP and health whilst considering potential confounding of neuroticism and familial influences. Individuals higher in FP had a lower risk of developing all studied diagnoses. Although neuroticism had considerable confounding effects on most flow-health relationships, higher FP significantly predicted a decreased risk for depression and anxiety even after adjusting for neuroticism and further adjustment for potential reverse causality. Lastly, when controlling for familial confounding, effect sizes for the associations between FP and both depression and anxiety remained similar, although they were no longer significant for anxiety when also adjusting for neuroticism. These findings are in line with a causal relation between proneness to flow experiences and depression and possibly anxiety.

### The association between flow proneness and the risk of health diagnoses

Across all diagnoses, higher FP significantly predicted a lower risk for developing mental and somatic health problems. This effect was moderately-strong and largest for depression and anxiety, where for every additional point on the FP scale, individuals were 16% less likely to be diagnosed. The effect was weakest for cardiovascular diseases: every point higher in FP resulted in a 5% lower risk of diagnosis. Reverse causality - that is, already acquired diagnoses precluding the experience of flow - seemed an unlikely explanation for most of these findings. FP remained a significant predictor for a diagnosis of depression and anxiety in the sensitivity analyses only including individuals who were first diagnosed after FP was measured in 2012. Despite the association between FP and stress disorders becoming non-significant, the HR remained similar in size, suggesting that the lack of statistical significance reflected loss of power due to the reduced sample size. Only the effects of FP on cardiovascular diseases diminished. It is conceivable that stress or the physical limitations that arise from a cardiovascular diagnosis prohibit engaging in flow-promoting activities [55–57], thus affecting FP.

### The confounding effect of neuroticism in the flow-health relationship

Given our finding that FP and phenotypic neuroticism are negatively related, we adjusted our survival models for neuroticism. As expected, neuroticism was associated with a higher risk of every diagnosis. Further, associations between FP and health diagnoses diminished (HRs moved closer to one) when adjusting for neuroticism, and this change was significant for depression, anxiety and bipolar and stress-related disorders. This suggests that the observed associations between flow and these health diagnoses are at least partially explained by neuroticism, with neuroticism possibly reducing the likelihood to experience flow and increasing the risk for health problems. Further testing showed that the genetic predisposition to neuroticism is significantly associated with lower FP. This suggests that genetic factors which underlie neuroticism influence FP, and the confounding effect of neuroticism observed may be (partially) due to shared genetics between FP and neuroticism.

Neuroticism is known to affects one’s predisposition to anxiety, as well as indirectly impacting anxiety and depression via increasing rumination and worry [58]. Though some studies suggest that neuroticism is a broader way of describing mood and anxiety disorders [59, 60], our results support the idea that neuroticism is only part of these conditions [61–63]. Most importantly, despite confounding effects of neuroticism, FP remained associated with a lower risk for depression and anxiety, with a trend towards significance for cardiovascular diseases (*p* = 0.06), indicating that neuroticism cannot explain the entire association between flow and the (mental) health conditions. For schizophrenia, bipolar and stress disorders, the effect of FP became non-significant, suggesting that neuroticism explained these flow-health associations. Together these findings highlight the importance for future studies to control for neuroticism when exploring effects of flow on health outcomes.

### Possible causal effects of flow proneness on the risk for health diagnoses

To control for familial confounding and strengthen causal inference, we examined differences in mental and somatic health diagnoses within monozygotic twin pairs discordant for FP. Monozygotic co-twins with higher FP had a significantly lower risk of receiving a depression diagnosis than their co-twin with lower flow, with the effect size remaining the same as seen in the full sample analyses, indicating a 16% (13% when also adjusting for neuroticism) lower risk for depression for every additional point on the FP scale. These findings suggest that familial confounding plays a negligible role in the association between flow proneness and depression and provide further support for a causal association, extending previous results [30] which reported associations between FP and low self-reported depressive symptoms after accounting for familial confounding (but not for neuroticism).

Interestingly, though non-significant when also adjusting for neuroticism, the size of the effect of FP on anxiety remained similar in the co-twin control analyses, indicating a 10% lower risk for developing an anxiety disorder for every additional point on the FP scale, after controlling for familial confounding. This suggests that FP is potentially also preventative of anxiety problems, but that the effect may not reach significance in the co-twin control analyses due to reduced sample size and, consequently, reduced power compared to the full-sample analyses (see limitations). This appears plausible also considering the comorbidity, overlapping symptomatology, and shared familial factors between depression and anxiety [64, 65]. Results for cardiovascular and stress models were non-significant. Even though we should be cautious with interpreting null-findings in the light of decreased power in co-twin control analyses, these findings highlight the importance to consider familial confounding when unraveling the link between health diagnoses and FP, particularly given the strong genetic components of both outcomes [66–68].

The present study is the first one to explore effects of FP on several mental and somatic health diagnoses and take confounding by family factors and neuroticism into account. Our findings lend further support to past research suggesting that flow experiences may protect against anxious and sad moods (e.g., [12, 24]), and therefore act as a protective factor against the risk of depression over time. Alternatively, as flow experiences have been shown to prevent rumination and promote distraction from maladaptive thinking [25, 26], perhaps flow prone individuals avoid persistent negative automatic thoughts that help instigate and maintain depression [69]. One of the critical features of the flow experience is an awareness of the present, specifically, a merging of action and awareness towards the task [26]. This is intrinsically preventative of dwelling on the past or worrying about the future, cognitions common in depression and anxiety [70–72]. Flow has also been shown to be associated with feelings of joy and mastery, which tend to be low in depressed individuals [73, 74]. Perhaps those who engage in flow more often experience fewer feelings of anhedonia and hopelessness in their everyday activities. Indeed, feeling accomplished and masterful (as autotelic individuals often do; [75]) might create a generalized optimism that prevents depressive states [76, 77]. Therefore, it is possible that interventions with flow-promoting components could have preventive effects for individuals at risk of developing depression.

#### Limitations and future directions

There are limitations within this study. The flow proneness measure was cross-sectional and undiagnosed health problems were obviously not accounted for. Furthermore, the outpatient registry only reached full coverage in 2001, i.e., some individuals with health problems before 2001 may not have a registered diagnosis. As such, we cannot fully exclude reverse causality, even in the co-twin control and post-2012 analyses, as some twins may have had sub-clinical concerns resulting in lower FP, which then later led to a diagnosis.

Second, we did not study comorbidity, receiving diagnoses multiple times, or diagnosis severity. Nonetheless, analyzing diagnoses provides a more objective, universal approach to mental and somatic health symptoms compared to self-report measures, which are prone to rater and recall biases. Third, as already discussed, statistical power may be an issue for some analyses, in particular the sensitivity analyses on reverse causality and the co-twin control analyses. Analyzing complete monozygotic twin pairs discordant on FP led to a decrease in sample size and therefore a decrease in the number of individuals who received a diagnosis. The same holds for the sensitivity analyses only including diagnoses obtained post 2012. The power of a method to analyze survival time data depends partly on the number of psychiatric diagnoses rather than on the total sample size. We should therefore be cautious to interpret null findings, and be conscious of the reduced power in the co-twin control analyses more generally. In line with this, it is important to note that we did not adjust for multiple testing, as we only test one overarching hypothesis (i.e., that flow proneness is related to registry-based diagnoses). There is no clear agreement as in how to adjust *p*-values in the case, where a Bonferroni correction is not appropriate. For this reason, actual *p*-values (rather than significance levels) have been reported throughout. However, we would like to point out that a few associations would not survive if we applied a more conservative *p*-value, highlighting the importance of future studies replicating our findings.

Fourth, significant associations in the co-twin control models may be attributable to other unmeasured confounds not shared within twins, but related to FP and mental health, such as twin pair differences in education. However, preliminary analyses on education as a confounder (see Appendix D, Table 4), which demonstrate a small, yet significant correlation between education level and FP (*r* = 0.03, *p* = 0.006), showed limited confounding effects in the association between FP and health.

Fifth, it is important to keep the conceptualization of ‘amount’ of FP in mind when interpreting the findings. We used the mean score of FP across several domains, which means that people who experience a lot of flow in one domain but not in others score the same as people who experience some flow in several domains. Future studies should compare health effects of flow in specific domains.

Last, response rates in the cohort have been low (60% in the first wave and even lower in further waves, i.e. around 38%). The low response rates are owed to the fact that this is a population based study (we are inviting a whole birth cohort of Swedish twins) of a working-aged cohort and are a general phenomenon not unique to our sample/study (see Zagai et al. [42]).

Though we did not investigate whether flow can be manipulated, the present findings give hope that flow interventions which promote flow experiences may help prevent depression and possibly anxiety disorders. Some studies suggests that mindfulness-based interventions increase flow in healthy individuals [78–80]. However, this is only investigated in the sports domain, and whether the findings would generalize to flow in other domains is unknown and it remains untested whether these interventions are effective in therapeutic contexts. The adaptive strategy of occupying the self through activity has served as an early form of ‘flow therapy’ for stress reduction [81]. Thus, a formalized activity therapy that attempts to induce the flow state may be the basis of intervention creation. Overall, it remains unknown as to how malleable flow is and whether any induced change in flow would also transfer to health effects. There are initial experiments that manipulate flow by creating conditions that supposedly elicit the flow experience [82–84]. Future studies should examine conditions to provoke flow experiences and investigate whether increases in flow experiences create the benefits outlined in the present study both in the general and a clinical population. The use of experience sampling methodologies may help to expand knowledge about the relationship between flow experiences and health over time. Finally, there may be a combination of several different pathways underlying the relationship between FP and mental health, including other possible confounders not studied here such as for example other personality traits, emotional intelligence [85], or socio-economic status etc. Future studies could explore such potential confounding factors further.

## Conclusion

The present study is the first to explore effects of flow proneness on several registry-based mental and somatic health diagnoses and to take confounding by family factors and neuroticism into account. Our findings provide novel evidence for a protective effect of FP on depression and potentially anxiety disorders and highlight the importance of controlling for neuroticism and familial confounding in research exploring potential positive health effects of flow experiences.

### Supplementary information

Appendices A to D

## Data Availability

The datasets generated during the current study cannot be made publicly as registry data were used. Individuals are able to apply online at the Swedish Twin Registry to access the twin data. Our analyses code is available at https://osf.io/haxt5/?view_only=225d3916d5ea4641801791654cae4091.
