# Supplementary material for: Can flow proneness be protective against mental and cardiovascular health problems? A genetically informed prospective cohort study
Source: Transl Psychiatry. 2024 Mar 13;14:144. doi: 10.1038/s41398-024-02855-6 (PMC10937942; doi:10.1038/s41398-024-02855-6)
Supplement: Supplementary file 1 — Appendices A to D [file 41398_2024_2855_MOESM1_ESM.docx]

Appendix A

**Swedish Flow Proneness Questionnaire (SFPQ)**

The SFPQ - English translation. Items 2–8 are only answered by individuals who are currently employed

1. Are you professionally active? (Yes/ no; If no, go to item 9)

When you do something at work, how often does it happen that…

1. …you feel bored?
2. …it feels as if your ability to perform what you do completely matches how difficult it is?
3. …you have a clear picture of what you want to achieve, and what you need to do to get there?
4. …you are conscious of how well or poorly you perform what you are doing?
5. …you feel completely concentrated?
6. …you have a sense of complete control?
7. …what you do feels extremely enjoyable to do?

When you are doing household work or other routine chores (e.g., cooking, cleaning, shopping) how often does it happen that…

9–15 Identical to items 2–8.

When you do something in your leisure time, how often does it happen that…

16–22 Identical to items 2–8.

**Appendix B**

***ICD Codes***

|  | | | |
| --- | --- | --- | --- |
| *ICD Codes 1969 - Present* | | | |
| Disorder Group | ICD 8 (1969-1986) | ICD 9 (1987-1996) | ICD 10 (1997-) |
| Depression | 296.2; 300.4 | 296B; 300E; 311 | F32 - F39 |
| Anxiety (OCD excluded) | 300.00; 300.10; 300.20; 300.5; 300.6; 300.7; 300.88; 300.99 | 300A; 300B; 300C; 300F | F40 - F41 |
| Schizophrenia & Schizoaffective Disorder | 295.0 - 295.4; 295.6; 295.8; 295.9 | 295A-295E; 295G; 295H; 295W; 295X | F20; F25 |
| Bipolar disorders | 296.0 - 296.3; 296.8; 296.9 | 296A-296E; 296W; 296X | F30 - F31 |
| Cardiovascular diseases | 390 - 589.99 | 390 - 459 | I00 - I99 |
| Stress disorders | 307; 308.4 | 308; 309 | F43 |
| *Note.* ICD = International Statistical Classification of Diseases and Related Health Problems; OCD = obsessive-compulsive disorder | | | |

Appendix C

**Swedish BFI-44 (adapted from Zakrisson, 2010) – English translation**

The following items are from the neuroticism subscale of the full Swedish BFI-44 (48). Reverse scored items are indicated by this symbol: (r)

I see myself as someone who…

4) Is depressed, down

9) Is relaxed, handles stress well (r)

14) Can be tense

19) Worries a lot

24) Is emotionally stable, does not get upset so easily (r)

29) Can be moody

34) Remains calm in tense situations (r)

39) Gets nervous easily

**Appendix D**

**Tables of Raw Hazard Ratios for all Analyses Performed**

| **Table 1****.** Hazard Ratios (HR) for the associations between flow proneness and risk of diagnoses, unadjusted (left) and adjusted (right) for neuroticism. | | | | | | | | | |
| --- | --- | --- | --- | --- | --- | --- | --- | --- | --- |
|  | | | | | | | | | |
|  | | Unadjusted for Neuroticism  (N_sample_ = 9,340) | | | | Adjusted for Neuroticism  (N_sample_ = 8,965) | | | |
| Diagnosis |  | *N_diagnosed_* | HR | 95% CI | *p* value | *N_diagnosed_* | HR | 95% CI | *p* value |
| Depression | Flow Proneness | 495 | 0.84 | 0.82 – 0.86 | <.001 | 475 | 0.94 | 0.91 – 0.97 | <.001 |
|  | Sex |  | 1.98 | 1.62 – 2.41 | <.001 |  | 1.50 | 1.22 – 1.84 | <.001 |
|  | Neuroticism |  | - | - | - |  | 3.47 | 3.01 – 4.00 | <.001 |
| Anxiety | Flow Proneness | 428 | 0.84 | 0.82 – 0.87 | <.001 | 402 | 0.95 | 0.92 – 0.99 | <.01 |
|  | Sex |  | 2.04 | 1.64 – 2.55 | <.001 |  | 1.49 | 1.18 – 1.87 | .001 |
|  | Neuroticism |  | - | - | - |  | 4.09 | 3.51 – 4.77 | <.001 |
| Schizophrenia | Flow Proneness | 27 | 0.85 | 0.75 – 0.96 | <.01 | 27 | 0.93 | 0.81 – 1.06 | .30 |
|  | Sex |  | 0.69 | 0.33 – 1.44 | .32 |  | 0.53 | 0.24 – 1.13 | .10 |
|  | Neuroticism |  | - | - | - |  | 2.86 | 1.60 – 5.07 | <.001 |
| Bipolar disorders | Flow Proneness | 91 | 0.88 | 0.82 – 0.94 | <.001 | 88 | 1.02 | 0.95 – 1.09 | .55 |
|  | Sex |  | 1.30 | 0.83 – 2.02 | .24 |  | 0.89 | 0.57 – 1.37 | .59 |
|  | Neuroticism |  | - | - | - |  | 4.64 | 3.34 – 6.44 | <.001 |
| Cardiovascular diseases | Flow Proneness | 439 | 0.96 | 0.92 – 0.99 | <.01 | 424 | 0.97 | 0.93 – 1.00 | .07 |
|  | Sex |  | 0.93 | 0.76 – 1.13 | .48 |  | 0.91 | 0.74 – 1.11 | .35 |
|  | Neuroticism |  | - | - | - |  | 1.20 | 1.01 – 1.40 | .03 |
| Stress disorders | Flow Proneness | 300 | 0.91 | 0.88 – 0.95 | <.001 | 285 | 1.00 | 0.96 – 1.05 | .69 |
|  | Sex |  | 1.87 | 1.44 – 2.41 | <.001 |  | 1.56 | 1.19 – 2.06 | .001 |
|  | Neuroticism |  | - | - | - |  | 2.83 | 2.34 – 3.41 | <.001 |
| ***Note.*** *N_sample_ =* number of individuals contributing to the analysis; *N_diagnosed_ =* number of cases (i.e., those diagnosed with subject condition); HR = hazard ratio; CI = confidence interval. | | | | | | | | | |

| **Table 2.** Hazard Ratios (HR) for the associations between flow proneness and risk of diagnoses, unadjusted and adjusted for neuroticism from the post 2012 sensitivity analyses. | | | | | | | | | |
| --- | --- | --- | --- | --- | --- | --- | --- | --- | --- |
|  | | | | | | | | | |
|  | | Unadjusted for neuroticism | | | | Adjusted for neuroticism | | | |
| Diagnosis (*N_sample)_* |  | *N_diagnosed_* | HR | 95% CI | *p* value | *N_diagnosed_* | HR | 95% CI | *p* value |
| Depression (8,972) | Flow Proneness | 126 | 0.84 | 0.79 – 0.89 | <.001 | 121 | 0.93 | 0.87 – 0.98 | .01 |
|  | Sex |  | 1.66 | 1.14 – 2.43 | <.01 |  | 1.31 | 0.89 – 1.94 | .51 |
|  | Neuroticism |  | - | - | - |  | 3.58 | 2.69 – 4.76 | <.001 |
| Anxiety  (9,036) | Flow Proneness | 124 | 0.84 | 0.79 – 0.89 | <.001 | 115 | 0.93 | 0.86 – 0.99 | .03 |
|  | Sex |  | 1.88 | 1.27 – 2.77 | .001 |  | 1.55 | 1.02 – 2.36 | .04 |
|  | Neuroticism |  | - | - | - |  | 3.65 | 2.76 – 4.83 | <.001 |
| Cardiovascular diseases  (9,084) | Flow Proneness | 185 | 1.00 | 0.95 – 1.05 | .97 | 177 | 1.02 | 0.97 – 1.07 | .44 |
|  | Sex |  | 0.78 | 0.58 – 1.03 | .08 |  | 0.74 | 0.54 – 1.00 | .05 |
|  | Neuroticism |  | - | - | - |  | 1.31 | 1.02 – 1.67 | .03 |
| Stress disorders  (9,145) | Flow Proneness | 103 | 0.95 | 0.89 – 1.02 | .14 | 99 | 1.05 | 0.97 – 1.12 | .20 |
|  | Sex |  | 2.22 | 1.40 – 3.52 | .001 |  | 1.85 | 1.13 – 3.00 | .01 |
|  | Neuroticism |  | - | - | - |  | 3.03 | 2.23 – 4.12 | <.001 |
| ***Note.*** *N_sample_ =* number of individuals contributing to the analysis (i.e., those not diagnosed with subject condition before 2012); *N_diagnosed_ =* number of cases (i.e., those diagnosed with subject condition after 2012); HR = hazard ratio; CI = confidence interval. | | | | | | | | | |

| **Table 3.** Hazard Ratios (HR) for the associations between flow proneness and risk of diagnosis, unadjusted and adjusted for neuroticism from the co-twin control analyses in monozygotic pairs. | | | | | | | | | |
| --- | --- | --- | --- | --- | --- | --- | --- | --- | --- |
|  | | | | | | | | | |
|  | | Unadjusted for Neuroticism | | | | Adjusted for Neuroticism | | | |
| Diagnosis |  | *N_pairs_* | HR | 95% CI | *p* value | *N_pairs_* | HR | 95% CI | *p* value |
| Depression | Flow Proneness | 97 | 0.84 | 0.74 – 0.95 | <.01 | 96 | 0.87 | 0.76 – 0.99 | .04 |
|  | Neuroticism |  | - | - | - |  | 1.86 | 1.02 – 3.39 | .04 |
| Anxiety | Flow Proneness | 79 | 0.87 | 0.76 – 0.99 | .03 | 76 | 0.90 | 0.77 – 1.03 | .12 |
|  | Neuroticism |  | - | - | - |  | 1.17 | 0.62 – 2.19 | .62 |
| Cardiovascular Diseases | Flow Proneness | 68 | 0.98 | 0.86 – 1.10 | .68 | 63 | 0.95 | 0.82 – 1.08 | .43 |
|  | Neuroticism |  | - | - | - |  | 0.79 | 0.37 – 1.63 | .52 |
| Stress disorders | Flow Proneness | 57 | 1.02 | 0.89 – 1.17 | .77 | 55 | 1.03 | 0.89 – 1.19 | .69 |
|  | Neuroticism |  | - | - | - |  | 1.75 | 0.83 – 3.66 | .14 |
| ***Note.*** *N_pairs_ =* number of twin pairs contributing to the analysis; HR = hazard ratio; CI = confidence interval; analyses are inherently adjusted for age and sex as monozygotic pairs by default are the same age and sex. | | | | | | | | | |

| Table 4a. Education adjusted models: Hazard Ratios (HR) for the associations between flow proneness and risk of diagnosis, unadjusted and adjusted for neuroticism. | | | | | | | | | |
| --- | --- | --- | --- | --- | --- | --- | --- | --- | --- |
|  | | Unadjusted (N_sample_ = 7,534) | | | | Adjusted (N_sample_ = 7,534) | | | |
| Diagnosis |  | *N_diagnosed_* | HR | 95% CI | *p* value | *N_diagnosed_* | HR | 95% CI | *p* value |
| Depression | Flow Proneness | 409 | 0.84 | 0.81 – 0.86 | <.001 | 409 | 0.93 | 0.89 – 0.96 | <.001 |
|  | Sex |  | 1.98 | 1.62 – 2.41 | <.001 |  | 1.55 | 1.23 – 1.94 | <.001 |
|  | Education |  | 1.02 | 0.97 –1.07 | .405 |  | 1.02 | 0.97 –1.07 | .306 |
|  | Neuroticism |  | - | - | - |  | 3.27 | 2.80 – 3.81 | <.001 |
| Anxiety | Flow Proneness | 334 | 0.84 | 0.81 – 0.87 | <.001 | 334 | 0.95 | 0.91 – 0.99 | .007 |
|  | Sex |  | 2.18 | 1.68 – 2.82 | <.001 |  | 1.57 | 1.21 – 2.04 | <.01 |
|  | Education |  | 0.95 | 0.90 –1.00 | .07 |  | 0.96 | 0.90 –1.01 | .11 |
|  | Neuroticism |  | - | - | - |  | 3.96 | 3.35 – 4.68 | <.001 |
| Schizophrenia | Flow Proneness | 27 | 0.85 | 0.75 – 0.96 | .01 | 26 | 0.93 | 0.81 – 1.07 | .30 |
|  | Sex |  | 0.78 | 0.36 – 1.64 | .51 |  | 0.61 | 0.28 – 1.32 | .21 |
|  | Education |  | .89 | 0.75 –1.05 | .18 |  | 0.90 | 0.76 –1.06 | .20 |
|  | Neuroticism |  | - | - | - |  | 2.70 | 1.50 – 4.84 | .001 |
| Bipolar disorders | Flow Proneness | 72 | 0.88 | 0.81 – 0.95 | <.01 | 72 | 1.02 | 0.94 –1.10 | .67 |
|  | Sex |  | 1.28 | 0.78 – 2.09 | .32 |  | 0.90 | 0.56 –1.45 | .68 |
|  | Education |  | 1.01 | 0.90 –1.11 | .92 |  | 1.01 | 0.91 –1.13 | .74 |
|  | Neuroticism |  | - | - | - |  | 4.45 | 3.08 – 6.40 | <.001 |
| Cardiovascular diseases | Flow Proneness | 360 | 0.96 | 0.92 – 0.99 | .02 | 360 | 0.97 | 0.93 – 1.01 | .19 |
|  | Sex |  | 1.02 | 0.82 – 1.27 | .85 |  | 0.99 | 0.79 – 1.23 | .90 |
|  | Education |  | .94 | 0.90 –0.99 | .02 |  | 0.95 | 0.90 –0.99 | .03 |
|  | Neuroticism |  | - | - | - |  | 1.19 | 1.00 – 1.41 | .05 |
| Stress disorders | Flow Proneness | 246 | 0.91 | 0.87 – 0.95 | <.001 | 246 | 1.00 | 0.96 – 1.05 | .76 |
|  | Sex |  | 1.98 | 1.48 – 2.63 | <.001 |  | 1.55 | 1.15 – 2.08 | <.01 |
|  | Education |  | 0.99 | 0.93 –1.05 | .74 |  | 1.00 | 0.94 –1.06 | .99 |
|  | Neuroticism |  | - | - | - |  | 2.88 | 2.36-3.52 | <.001 |

***Note.*** Nsample = number of individuals contributing to the analysis; Ndiagnosed = number of cases (i.e., those diagnosed with subject condition); HR = hazard ratio; CI = confidence interval.

| Table 4b. Education adjusted models: Hazard Ratios (HR) for the associations between flow proneness and risk of diagnosis, unadjusted and adjusted for neuroticism from the post 2012 sensitivity analyses. | | | | | | | | | |
| --- | --- | --- | --- | --- | --- | --- | --- | --- | --- |
|  | | | | | | | | | |
|  | | Unadjusted | | | | Adjusted | | | |
| Diagnosis (*N_sample)_* |  | *N_diagnosed_* | HR | 95% CI | *p* value | *N_diagnosed_* | HR | 95% CI | *p* value |
| Depression (7,224) | Flow Proneness | 99 | 0.82 | 0.77 – 0.87 | <.001 | 99 | 0.91 | 0.84 – 0.97 | <.01 |
|  | Sex |  | 1.72 | 1.11 – 2.64 | .01 |  | 1.32 | 0.85 – 2.03 | .22 |
|  | Education |  | 1.07 | 0.97 – 1.18 | .14 |  | 1.07 | 0.98 – 1.18 | .12 |
|  | Neuroticism |  | - | - | - |  | 3.37 | 2.44 – 4.64 | <.001 |
| Anxiety  (7,292) | Flow Proneness | 92 | 0.83 | 0.77 – 0.89 | <.001 | 92 | 0.93 | 0.85 – 1.00 | .07 |
|  | Sex |  | 2.36 | 1.43 – 3.87 | .001 |  | 1.79 | 1.09 – 2.94 | .02 |
|  | Education |  | 1.05 | 0.95 – 1.14 | .31 |  | 1.05 | 0.96 – 1.15 | .25 |
|  | Neuroticism |  | - | - | - |  | 3.56 | 2.59 – 4.88 | <.001 |
| Cardiovascular diseases  (7,319) | Flow Proneness | 148 | 1.00 | 0.94 – 1.05 | .92 | 148 | 1.02 | 0.96 – 1.08 | .47 |
|  | Sex |  | 0.96 | 0.69 – 1.33 | .82 |  | 0.91 | 0.65 – 1.27 | .58 |
|  | Education |  | 0.95 | 0.88 – 1.02 | .18 |  | 0.96 | 0.88 – 1.03 | .22 |
|  | Neuroticism |  | - | - | - |  | 1.33 | 1.01 – 1.74 | .04 |
| Stress disorders  (7,373) | Flow Proneness | 84 | 0.94 | 0.88 – 1.01 | .10 | 84 | 1.06 | 0.97 – 1.14 | .16 |
|  | Sex |  | 2.19 | 1.31 – 3.65 | <.01 |  | 1.68 | 1.00 – 2.80 | .05 |
|  | Education |  | 0.95 | 0.86 – 1.04 | .24 |  | 0.97 | 0.88 – 1.06 | .46 |
|  | Neuroticism |  | - | - | - |  | 3.36 | 2.39 – 4.71 | <.001 |
| *Note. N_sample_ =* number of individuals contributing to the analysis (i.e., those not diagnosed with subject condition before 2012); *N_diagnosed_ =* number of cases (i.e., those diagnosed with subject condition after 2012); HR = hazard ratio; CI = confidence interval | | | | | | | | | |

| Table 4c. Education adjusted models: Hazard Ratios (HR) for the associations between flow proneness and risk of diagnosis, unadjusted and adjusted for neuroticism from the co-twin control analyses in monozygotic pairs. | | | | | | | | | |
| --- | --- | --- | --- | --- | --- | --- | --- | --- | --- |
|  | | Unadjusted | | | | Adjusted | | | |
| Diagnosis |  | *N_pairs_* | HR | 95% CI | *p* value | *N_pairs_* | HR | 95% CI | *p* value |
| Depression | Flow Proneness | 76 | 0.79 | 0.67 – 0.92 | <.01 | 76 | 0.81 | 0.69 – 0.95 | <.01 |
|  | Education |  | 0.99 | 0.70 – 1.41 | .98 |  | 0.98 | 0.68 – 1.39 | .90 |
|  | Neuroticism |  | - | - | - |  | 1.74 | 0.84 – 3.59 | .13 |
| Anxiety | Flow Proneness | 55 | 0.86 | 0.73 – 1.02 | .08 | 55 | 0.86 | 0.72 – 1.02 | .09 |
|  | Education |  | 0.99 | 0.71 – 1.37 | .96 |  | 0.99 | 0.71 – 1.38 | .97 |
|  | Neuroticism |  | - | - | - |  | 0.97 | 0.42 – 2.19 | .93 |
| Cardiovascular Diseases | Flow Proneness | 49 | 0.91 | 0.77 – 1.07 | .27 | 49 | 0.92 | 0.77 – 1.09 | .35 |
|  | Education |  | 1.04 | 0.79 – 1.39 | .75 |  | 1.05 | 0.79 – 1.39 | .74 |
|  | Neuroticism |  | - | - | - |  | 1.11 | 0.50 – 2.46 | .79 |
| Stress disorders | Flow Proneness | 46 | 0.97 | 0.83 – 1.13 | .67 | 46 | 0.99 | 0.83 – 1.16 | .85 |
|  | Education |  | 0.78 | 0.53 – 1.15 | .21 |  | 0.77 | 0.52 – 1.15 | .21 |
|  | Neuroticism |  | - | - | - |  | 1.71 | 0.74 – 3.91 | .21 |
| *Note. N_pairs_ =* number of twin pairs contributing to the analysis; HR = hazard ratio; CI = confidence interval | | | | | | | | | |
